# Exploring the Antibacterial and Antifungal Potential of Jellyfish-Associated Marine Fungi by Cultivation-Dependent Approaches

**DOI:** 10.1371/journal.pone.0144394

**Published:** 2015-12-04

**Authors:** Yang Yue, Huahua Yu, Rongfeng Li, Ronge Xing, Song Liu, Pengcheng Li

**Affiliations:** 1 Key Laboratory of Experimental Marine Biology, Institute of Oceanology, Chinese Academy of Sciences, Qingdao, China; 2 University of the Chinese Academy of Sciences, Beijing, China; Universita degli Studi di Pisa, ITALY

## Abstract

Fungi isolated from marine invertebrates are of considerable importance as new promising sources of unique secondary metabolites with significant biomedical potential. However, the cultivable fungal community harbored in jellyfish was less investigated. In this work, we seek to recover symbiotic fungi from different tissues of jellyfish *Nemopilema nomurai*. A total of seven morphotypes were isolated, which were assigned into four genera (*Aspergillus*, *Cladosporium*, *Purpureocillium*, and *Tilletiopsis*) from two phyla (Ascomycota and Basidiomycota) by comparing the rDNA-ITS sequences with the reference sequences in GenBank. The most fungi were found in the inner tissues of subumbrella. Two of the cultivation-independent procedures, changing media type and co-cultivation, were employed to maximize the complexity of metabolites. Thus, thirteen EtOAc gum were obtained and fingerprinted by High Performance Liquid Chromatography (HPLC) equipped with a photodiode array (PDA) detector. Antibacterial and antifungal activities of these complex mixtures were tested against a panel of bacterial and fungal pathogens. The antimicrobial results showed that all of the 13 EtOAc extracts displayed different levels of antibacterial activity, three of which exhibited strong to significant antibacterial activity to the bacterial pathogens *Staphylococcus aureus* and *Salmonella entrica*. Antifungal activity indicated that the EtOAc extracts from pure culture of *Aspergillus versicolor* and co-culture of *A*. *versicolor* and *Tilletiopsis* sp. in rice media were promising for searching new compounds, with the maximal mycelial growth inhibition of 82.32% ± 0.61% for *Rhizoctonia solani* and 48.41% ± 11.02% for *Botrytis cinerea* at 200 μg/ml, respectively. This study is the first report on the antibacterial and antifungal activity of jellyfish-associated fungi and allows the first sight into cultivable fungal community residing in jellyfish. Induced metabolites by cultivation-dependent approaches provides a new reservoir for drug discovery from jellyfish-derived fungi.

## Introduction

Jellyfishes belong to the phylum Cnidaria, and at least four toxic classes are included in this phylum, namely Anthozoa, Cubozoa, Scyphozoa and Hydrozoa [1]. Medusa of the class Scyphozoa are usually referred to true jellyfishes, and three representatives of this extraordinary class, *Aurelia aurita*, *Cyanea nozaki* and the giant *N*. *nomurai* cause serious damage, inducing outbreaks in China’s coastal waters, threats to fishery, and coastal facilities and even death of tourists for stinging. Research has been focused by scientists on ecosystem effect of jellyfish blooms, life cycle and toxic effects of venom and toxins. Recently, an interesting research suggested that jellyfish ancestor may have got a *pgsAA* gene essential for PGA synthesis during nematocyte formation from a prokaryote by horizontal gene transfer (HGT) [2], indicating a close relationship between jellyfish ancestors and bacteria.

Marine chemists seek to find bioactive secondary metabolites from diverse marine habitats, including coastal coral-reef, mangrove peat, deep sea and symbionts of marine invertebrates. Symbiosis has proven to be a source of highly diverse compounds [3–5]. In some cases, marine microorganisms, especially marine symbiotic bacteria, are thought to be the true producers of some active metabolites found in marine invertebrates [6, 7]. Interestingly, jellyfish was observed to have diverse symbiotic relationships with various creatures, ranging from macroorganisms such as shrimp [8] and crustaceans [9, 10] to microorganisms such as dinoflagellate[11, 12]. Endobiotic bacteria were also detected in the tentacles of jellyfish species *Cyanea capillata* and *Cyanea lamarckii* [13]. Likewise other marine invertebrates such as sponges and corals, which host a variety of symbiotic fungi or actinomycetes, jellyfish-associated microorganisms may be an untapped source of new marine natural products.

Historically, the search for small-molecule natural products from jellyfish-associated microorganisms has been neglected by marine natural products chemists. Only few studies have been focused on isolation of secondary metabolites from jellyfish-associated actinomycete [14, 15], dinoflagellate [16] and fungi [17, 18]. In contrast to the association between jellyfish and obligately symbiotic dinoflagellate, very little is known about the fungal community in jellyfish. To our knowledge, there are only 4 jellyfish-derived fungi reported in literatures [17–19], which account for a very tiny slice of fungi isolated from marine invertebrates. Although it’s still hard to determine the true identity of fungi harbored in jellyfish, exploiting the fungal resources in view of developing agricultural antibiotics remains urgent. The venomous jellyfish *N*. *nomurai* [20], a synonym of *Stomolophus meleagris* in our former publications [21–24], is a commonly reoccurring specimen during summer in Qingdao. In the present study, we describe the isolation and identification of fungi from different tissues of the jellyfish *N*. *nomurai*. One Strain-Many Compounds (OSMAC) strategy [25, 26] and co-cultivation of strains were employed to maximize discovery of metabolically bioactive and diverse compounds. EtOAc extracts of OSMAC and co-cultures were further characterized by HPLC-PDA analysis and finally used for screening of antibacterial and antifungal activity against six bacterial pathogens and five fungal pathogens. Given that, at the best of our knowledge, there are few reports about the isolation of cultivable jellyfish-associated fungi, this is possibly the first report in which different fungal taxa have been associated to different jellyfish tissues and that unveils the potential of fungi of jellyfish subumbrella for the discover of new antimicrobial and antifungal agents.

## Results and Discussion

### Isolation and identification of the symbiotic fungi from jellyfish inner tissues of Umbrella, Tentacle and Gonad

**Fig 1:**
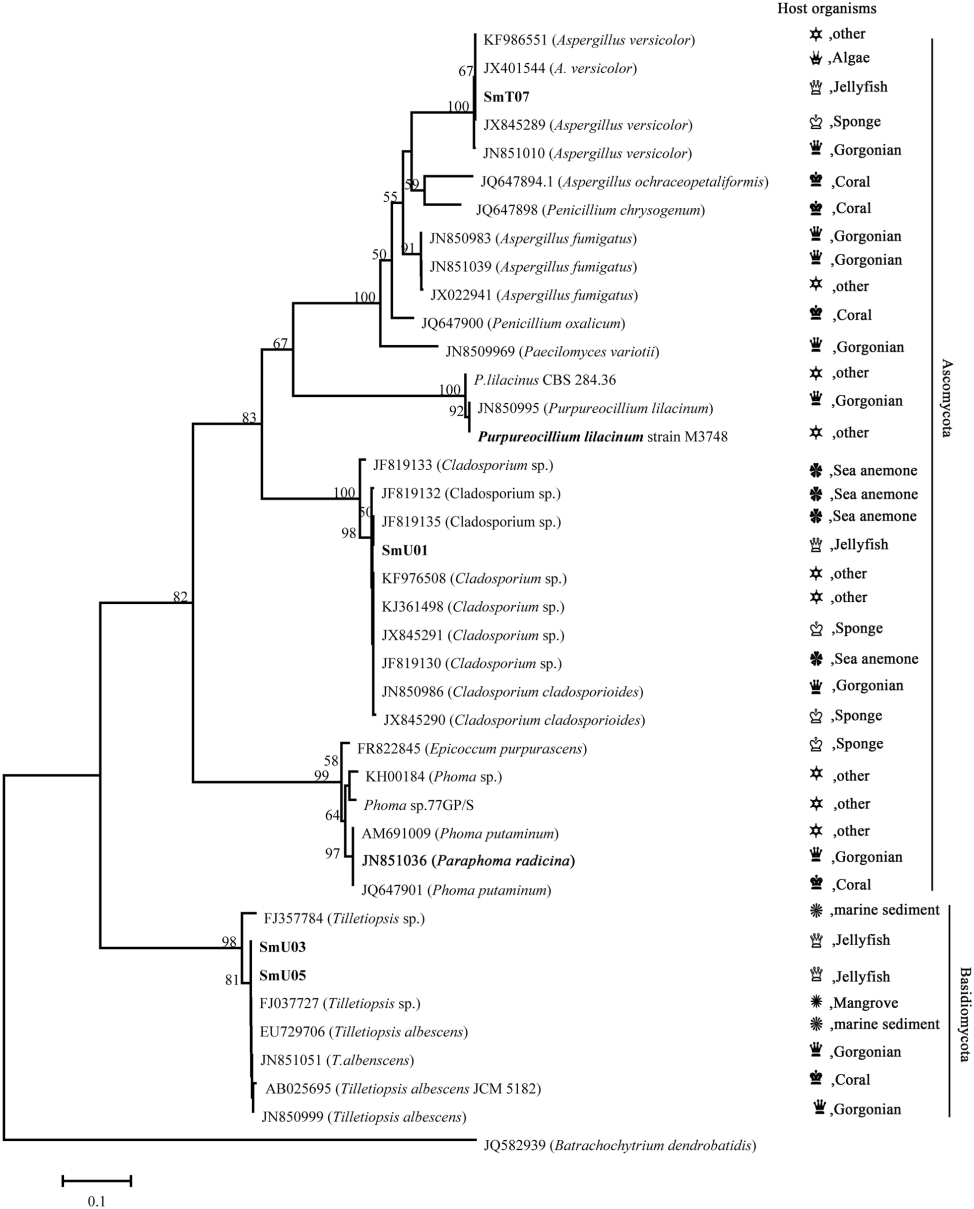
Neighbor-joining phylogenetic tree from analysis of available rDNA-ITS sequences of all identified jellyfish-derived fungi. The ITS sequences obtained in this work were indicated in bold. Bootstrap values were indicated at nodes of each branch based on a neighbor-joining analysis of 1000 replicates. Only values of > 50% were shown. Scale bar was equal to 0.1 substitutions per nucleotide position.

In order to determine which parts of the jellyfish *N*. *nomurai* may host cultivable fungi, representative tissues of umbrella, tentacle and gonad were sampled and inoculated on the isolation media with two different methods. The total number of fungal colonies observed on PDA plates was 7, which were further transferred to new PDA plates to get pure cultures. Of all the fungal morphotypes isolated, only 2 isolates (SmT06, SmT07) were recovered from the inner tissues of oral arm adjoining to the long tentacles. The remaining 5 strains (SmU01~SmU05) were isolated from sub-umbrella. However, plates containing inner tissues of Exumbrella and Gonad yielded no isolates. Excluding the redundant strains with identical morphological characteristics, 5 isolates (Table 1, Fig 1) were sequenced and identified at molecular level. By comparison with sequences in GenBank, isolates sharing 99~100% similarity with their closest NCBI relatives were assigned to two fungal phyla: Ascomycota and Basidiomycota. Except for 3 isolates (SmU03~SmU05) of the genus *Tilletiopsis* in phylum Basidiomycota, the remaining 4 isolates distributed in 3 genera of phylum Ascomycota: *Cladosporium* (SmU01), *Purpureocillium* (SmU02), *Aspergillus* (SmT06, SmT07). The widest diversity of fungi were found in the inner tissues of Subumbrella.

**Table 1:** Identification of fungal strains isolated from the jellyfish samples based on morphological characteristics as well as DNA analysis of the internal transcribed spacer (ITS) region. The closest relatives in GenBank according toBLAST search were presented.

Isolates	Host Tissue	Seq. Length (bp)	Access Number	The Closest Strains	Similarity (%)	Overlap (bp)
SmU0 (Y1)	sub-umbrella	464	KP081770	*Cladosporium* sp.	99	463
SmU02 (Y2)	sub-umbrella	541	KP081171	*Purpureocillium lilacinum*	99	540
SmU03 (Y3)	sub-umbrella	581	KP081172	*Tilletiopsis albescens*	100	581
SmU05^a^ (Y5)	sub-umbrella	565	KP081173	*Tilletiopsis* sp.	99	563
SmT07^b^ (Y11)	tentacle	481	KP081174	*Aspergillus versicolor*	100	481

^a^: isolate SmU04 was identical to SmU05, according to our preliminary screening of redundant strains;

^b^: isolate SmT06 was identical to SmT07, according to our preliminary screening for redundant strains.

The diversity of fungi recovered from jellyfish was far less than that isolated from its benthic cnidarian counterparts such as corals and sea anemones. This can be partially explained by the theory of chemical defense. In hostile marine habitats, benthic invertebrates lacking physical protection produce unusual secondary metabolites to protect themselves from predators, while jellyfishes usually deter predation by their formidable venom [27, 28]. Another negative factor leading to low recovery of fungi is the high water content in jellyfish tissues [29]. Although failing to isolate fungi from inner tissues of Tentacle and Gonad, these parts are thought to be rich in bacteria [13, 30–32]. To allow for a deeper insight into fungal community associated with jellyfish, a neighbor-joining phylogenetic tree (Fig 1) was reconstructed. All the ITS sequences used for phylogenetic analysis were listed in S1 Table. The results of rDNA-ITS sequences analysis showed that the phylum Ascomycota dominate, which is in accordance with other studies on marine invertebrate-derived fungi [33–35]. Most jellyfish-derived fungi (9 isolates, 7 distinct ribotypes) belonged to the phylum Ascomycota and were distributed among 5 different orders and 6 genera (Fig 1): Capnodiales (*Cladosporium*); Eurotiales (*Aspergillus* and *Byssochlamys*); Hypocreales (*Purpureocillium*); Pleosporales (*Phoma*); Georgefischeriales (*Tilletiopsis*). Additionally, *Epicoccum purpurascens*, isolated from the jellyfish *Aurelia auritav* was affiliated to the Class Dothideomycetes (*Epicoccum*) [19]. *Purpureocillium* sp. (strain SmU02) that belongs to the new established genus *Purpureocillium* [36] was also isolated and reported from jellyfish for the first time in this study. The phylum Basidiomycota was represented by 3 isolates affiliated to the genera *Tilletiopsis* (Fig 1). Although records occurred in deep-sea sediments [33] and gorgonians [37], it’s not common to recover this fungal species from marine environments. To our knowledge, *Tilletiopsis* spp. have been first isolated from jellyfish. The current data suggest that jellyfish could be an untapped source of marine-derived fungi. However much more efforts should be taken to unveil the fungal community inside jellyfish, since that over 250 species of jellyfish are distributed in the ocean and most of them have been scarcely investigated.

### HPLC-PDA profiles of the crude EtOAc extracts obtained by cultivation-dependent approaches

**Fig 2:**
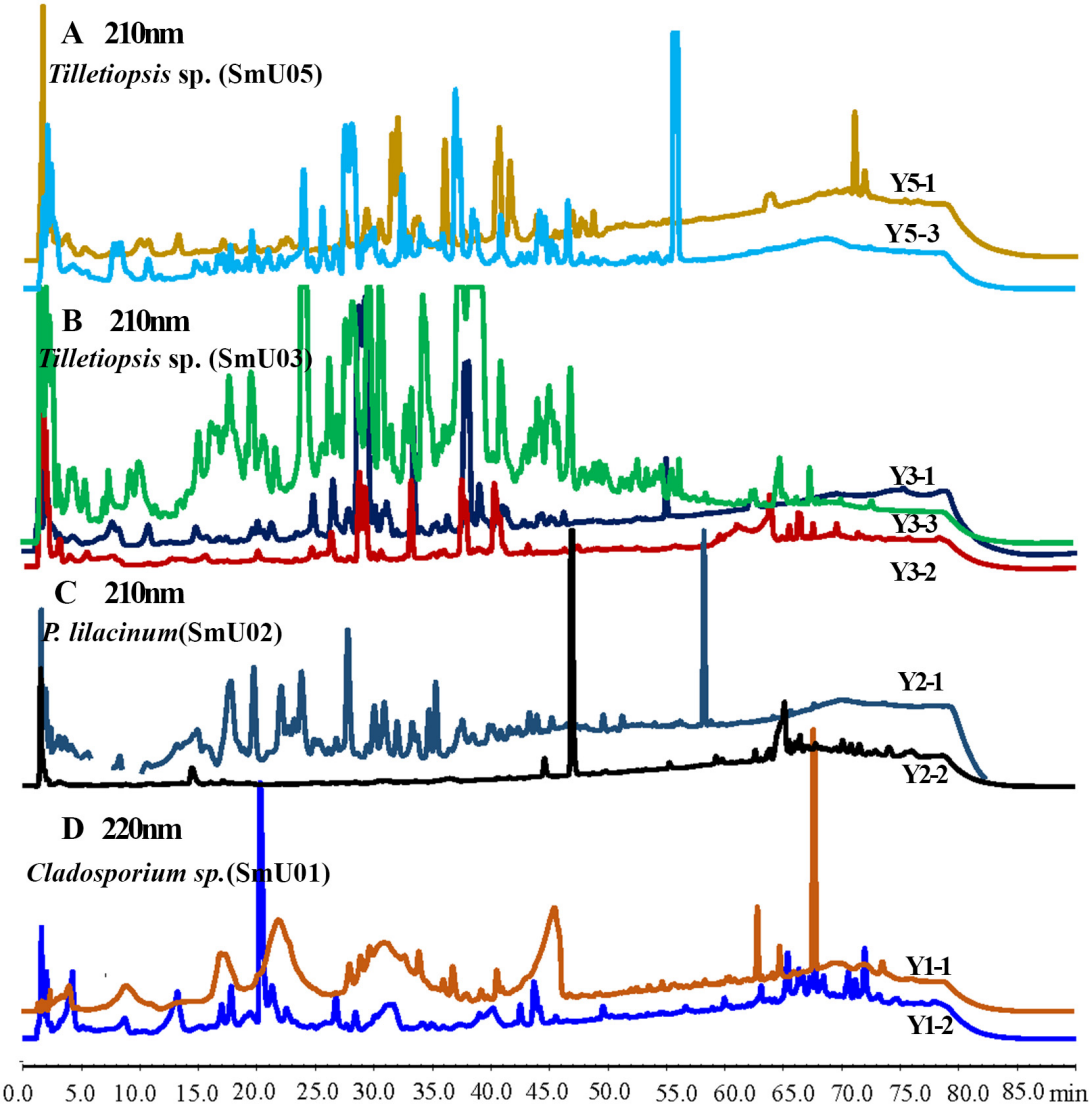
HPLC chromatograms of EtOAc extracts of (A) *Tilletiopsis* sp., (B)*Tilletiopsis albescens*, (C) *P*. *lilacinum*, (D) *Cladosporium* sp. cultivated with WMB, RM, ISP2 media detected at 210nm or 220nm.

By OSMAC and co-cultivation strategies, a total of 13 organic extracts were obtained as dark brown gum. All of them were listed in Table 2. To analyze the composition variations in different culture media, HPLC-PDA analysis was conducted. The HPLC fingerprints of *Tilletiopsis* spp. (strains SmU03 and SmU05) were given for the first time, and strain SmU03 showed enhanced chemical diversity when cultivated in ISP2 medium (Y3-3, green line, Fig 2). Among these chromatograms, the biggest change occurred in organic extract of rice solid culture of isolate SmU02 (Y2-2, black line, Fig 2), which yielded fewer peaks than Y2-1 (Table 2). These results demonstrated that the OSMAC strategy could effectively enhance the microbial biosynthetic potentials.

**Table 2:** Results of agar diffusion assays for the antibiotic activity of ethyl acetate (EtOAc) extracts from OSMAC cultures of the giant jellyfish-derived fungi.

Fungi	Media	EtOAc Extracts	Bacterial and fungal Pathogens							
			*S*. *aureus*	*E*. *coli*	*S*. *enterica*	*V*. *parahaemolyticus*	*L*. *pelagia*	*B*. *subtilis*	*C*. *albicans*	*A*. *niger*
SmU01 (Y1)	WMB	Y1-1	**++** ^a^	**-** ^c^	**++++** ^a^	**-** ^f^	**+** ^bc^	**+** ^cde^	**-**	**-**
	RM	Y1-2	**-** ^d^	**-** ^c^	**++++** ^a^	**-** ^f^	**+** ^bc^	**++** ^b^	**-**	**-**
SmU02 (Y2)	WMB	Y2-1	**++** ^a^	**++** ^a^	**+++** ^b^	**+** ^def^	**+** ^bc^	**++** ^a^	**-**	**-**
	RM	Y2-2	**++** ^a^	**-** ^c^	**++** ^c^	**-** ^f^	**+** ^bc^	**-** ^e^	**-**	**-**
SmU03 (Y3)	WMB	Y3-1	**+** ^b^	**+** ^b^	**+** ^d^	**++** ^a^	**++** ^abc^	**+** ^de^	**-**	**-**
	RM	Y3-2	**+** ^cd^	**-** ^c^	**++** ^cd^	**++** ^a^	**++** ^ab^	**+** ^cde^	**-**	**-**
	ISP2	Y3-3	**+** ^b^	**+** ^b^	**++** ^c^	**+** ^b^	**++** ^abc^	**+** ^cd^	**-**	**-**
SmU05 (Y5)	WMB	Y5-1	**+** ^bc^	**+** ^b^	**+++** ^b^	**+** ^bcd^	**++** ^ab^	**++** ^b^	**-**	**-**
	ISP2	Y5-3	**+** ^b^	**+** ^b^	**++** ^cd^	**+** ^cde^	**-** ^c^	**+** ^bc^	**-**	**-**
SmT07 (Y11)	WMB	Y11-1	**-** ^d^	**-** ^c^	**-** ^e^	**-** ^f^	**++** ^bc^	**+** ^cde^	**-**	**-**
	RM	Y11-2	**+** ^bc^	**+** ^b^	**-** ^e^	**+** ^bc^	**++** ^ab^	**+** ^cd^	**-**	**-**
Co-culture (CO)	WMB	CO-1	**+** ^bc^	**+** ^b^	**-** ^e^	**-** ^f^	**++** ^ab^	**+** ^cd^	**-**	**-**
	RM	CO-2	**+** ^bc^	**-** ^c^	**-** ^e^	**-** ^f^	**++** ^a^	**+** ^cd^	**-**	**-**
NC			**-**	**-**	**-**	**-**	**-**	**-**	**-**	**-**
PC			**++++**	**++++**	**++++**	**++++**	**++++**	**++++**	**+**	**++**

**NOTE:** “CO”, co-cultivate fungi SmU05 and SmT07; NC, negative control (DMSO); PC, positive control (Ciprofloxacin for bacteria, 16ug/disk; Amphotericin B for *C*. *albican and A*. *niger*, 12ug/disk). The diameter of inhibition zone (mm) was finally reported as:

“-”, no inhibition effect was detected;

“+”, the inhibition zone ranged in 7~9mm (weak);

“++”, between 10mm and 15mm (moderate);

“+++”, less than 20mm but more than 15mm (strong);

“++++”, more than 20mm (significant). Mean inhibition zone (mm) against each bacterial and fungal pathogen with different alphabets (a~f) within the same column are significantly different (p < 0.05).

**Fig 3:**
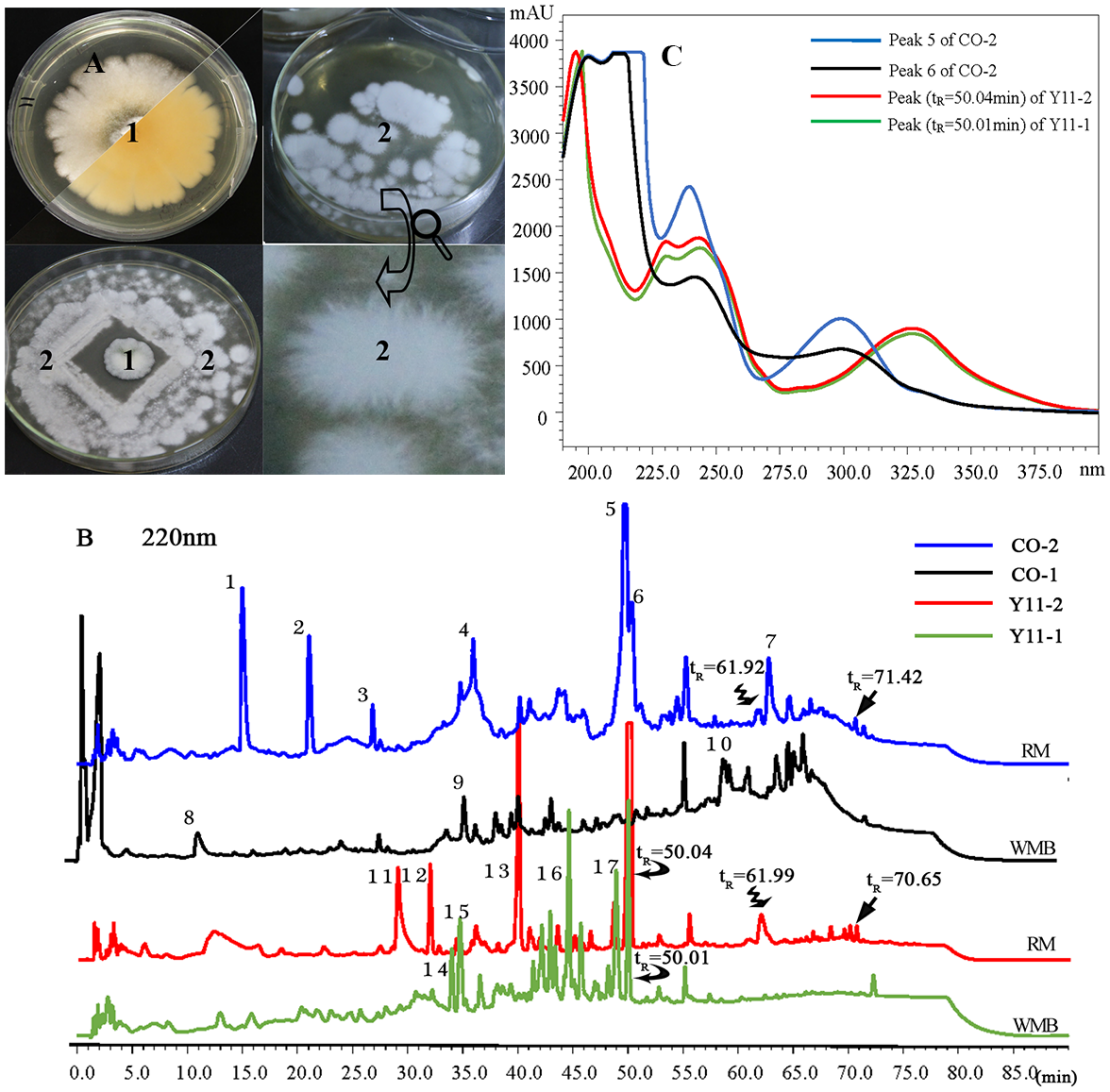
(A) Photograph of pure strains (1) *Aspergillus versicolor*, (2)*Tilletiopsis albescens* and co-cultivation of two jellyfish-derived fungi on Wickerham’ medium plates (Φ = 9cm). (B) HPLC chromatograms of EtOAc extracts of *Aspergillus versicolor* (Y11) and co-culture (CO) cultivated with WMB and RM medium were presented with different colored lines under 220nm. Peaks with the same retention time (t_R_) and UV absorbance spectra were labeled with different arrows. New peaks induced by varying media or co-cultivation were indicated with number 1~17 in each chromatogram. (C) UV absorbance spectra of peaks at t_R_ = 50min of Y11-1, Y11-2, CO-1 and CO-2.

Fungal-fungal co-culture assays were achieved by inoculating fungal strains together in a single agar plate to determine the morphological interaction pattern. The strain SmU05, *Tilletiopsis* sp., was able to inhibit strain SmT11, *A*. *versicolor*, at a long distance. After inoculating in the dark for two weeks, intriguing morphological appearance was detected when the mycelial growth of *A*. *versicolor* stopped at a distance from the fungal colony of *Tilletiopsis* sp. (Fig 3A). Historically, microbial co-culture usually confronted fungus with bacterium or fungus of different source in the same vessel [38, 39]. Recent isolation of new induced metabolites such as fumiformamide 1 [40] and 4”-hydroxysulfoxy-2, 2”-dimethylthielavin P [41] demonstrated that antagonistic microbial co-culture maybe useful for drug discovery. The morphological interaction reported here between *A*. *versicolor* and *Tilletiopsis* sp. is one of the few cases of co-cultivation of two antagonistic marine-derived fungi for induction of new metabolites. Given the prolific nature of *A*. *versicolor* in diverse secondary metabolites [42–44] and the still unknown metabolic potential of *Tilletiopsis* sp., this fungal interaction is of much interest.

The chemodiversity and variation in metabolite compositions between pure strains and the compared co-cultures were further revealed by HPLC-PDA analysis of their EtOAc crudes. Four representatives of HPLC chromatograms with UV absorbance spectra were shown in Fig 3B. By a close inspection into the HPLC chromatograms, we found several peaks at similar retention time (t_R_, indicated by different arrows in Fig 2B) of different chromatograms shared the identical UV absorbance pattern, indicating that similar metabolites were produced in different media or co-cultivation condition. The occurrence of same peaks was a very useful information for comparison of metabolites complexity, because they not only confirmed the repeatability of analytical condition, but also served as an efficient indicator to locate other different peaks. Noteworthy, peaks 5 and 6 appeared around t_R_ = 50min in chromatogram of CO-2 (blue line, Fig 3B) with a maximal UV absorbance at 300nm (Fig 3C), which were different to those peaks with a maximal UV absorbance at 325nm of Y11-1 and Y11-2. These two peaks were produced only by co-cultivation on rice solid medium while they were not detected in liquid co-culture of *A*. *versicolor* and *Tilletiopsis* sp.. Other new representative peaks in each chromatogram were indicated with number 1~17 in Fig 3B. The most enhanced productivity and chemodiversity was detected in EtOAc extract of CO-2.

### Antimicrobial and antifungal activities of EtOAc extracts of OSMAC and co-cultures of jellyfish-derived fungi

The obtained thirteen EtOAc extracts of OSMAC and co-cultures of five jellyfish-derived fungi were tested for antimicrobial activities against a panel of eight indicator strains, composed of six bacterial pathogens and two fungal indicator strains (C. *albicans* and A. *niger*) by an agar diffusion method. Results of antimicrobial activity were given in Table 2. All of the 13 tested samples exhibited different spectrum of antimicrobial activity against all of the bacterial pathogens, and the diameter of inhibition zone (mm) ranged from 7mm to 22mm. Compared with other crude extracts, both Y1-1 and Y1-2 showed the most significant inhibitory activity against *S*. *aureus*. Strong antibacterial activity of Y1-1 was also observed against Gram-positive bacteria *S*. *aureus*, while Y1-2 showing no inhibition against *S*. *aureus*. The counterparts of *Cladosporium* sp. (SmU01) from mangrove and deep sea have been reported to produce antiviral alkaloids [45] and polyketides [46], strain SmU01 (*Cladosporium* sp.) may hold potential in synthesizing new antimicrobial metabolites. Given the dramatic changes that occurred in HPLC-PDA chromatograms with antibacterial spectra, liquid Wickerham’ medium seems to be much more suitable for inducing bioactive compounds [29]. This phenomena was also observed in Y2-1, in which case Y2-1 showed weak to strong antibacterial activity against six bacterial indicators while Y2-2 only displayed moderate level of inhibition to *S*. *aureus* and *S*. *enterica* and weak inhibition to *L*. *pelagia* (Table 2). Although cultivated in three different media, isolate *Tilletiopsis albescens* showed almost same antimicrobial activities against all the bacterial indicators. And for the first time, *Tilletiopsis* spp. were found to possess antibacterial activities against human pathogens. Compared with Y1-1 and Y2-1, crude extracts from pure strains (SmU05, SmT07) and their co-cultures showed weak antimicrobial property except for *L*. *pelagia*. We must point out that no inhibition was detected to both fungal indicators C. *albicans* and A. *niger* among all the thirteen EtOAc extracts. The diameter of inhibition zone of amphotericin B at 12 μg /disk for C. *albicans* and A. *niger* was 9.2 ± 0.29 mm and 14.8 ± 1.0 mm, respectively.

**Fig 4:**
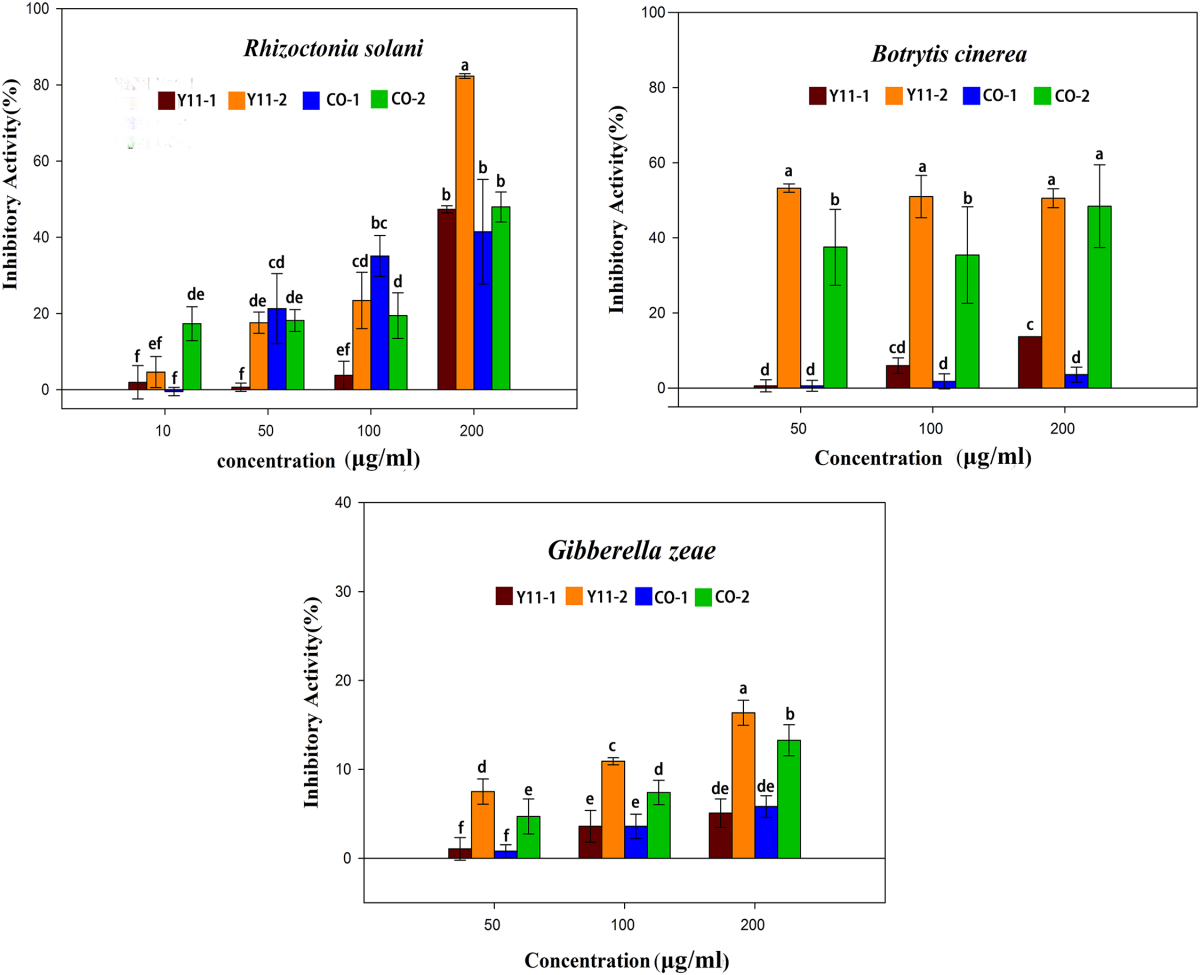
Results of antifungal activity of crude extracts from pure stain SmT07 and co-culture strains in liquid Wickerham’ medium and solid rice medium against three common phytopathogenic fungi. Bars indicate the standard deviation from four replicates. Different alphabets above the bars have mean values that are significantly different (p < 0.05).

In Table 2, Y11-2 and CO-2 were found to possess weak antibacterial activity, although observed with enhanced productivity and diversity in Fig 3B. We wondered if these induced EtOAc mixtures would lead to discovery of fungicides for agricultural purposes. So antifungal activities of co-cultures and pure cultures in different media were further evaluated against four plant pathogenic fungi, *Rhizoctonia solani* CGMCC 3.28, *Verticillium dahliae* CGMCC 3.3758, *Gibberella zeae* CGMCC 3.4288, *Botrytis cinerea* CGMCC 3.4582. Results of antifungal activities of four crude extracts (Y11-1, Y11-2, CO-1 and CO-2) were showed in Fig 4. These results indicated that Y11-2 exhibited a significant inhibitory activity at three concentrations tested (50, 100, 200 μg/ml), with a maximal inhibitory mycelial growth of 82.32% ± 0.61% against *R*. *solani* at 200 μg/ml and 53.23% ± 1.13% against *B*. *cinerea* at 50 μg/ml. CO-2 also showed a good antifungal potential against *R*. *solani* and *B*. *cinerea* at 200 μg/ml, and the inhibitory activity were 47.97% ± 3.92%, 48.41% ± 11.02%, respectively. All of the four crude extracts exhibited weak inhibition against *G*. *zeae* (Fig 4) and no inhibition against *V*. *dahliae* at three concentrations tested. Y11-2 and CO-2 will likely be good candidates for further isolation of agricultural fungicides due to their strong antifungal activities and significant chemical diversity.

## Experimental Section

### Sample Materials

In this study, collection of jellyfish samples was only referred to the collection of living specimen of jellyfish *N*. *nomurai*. In China, jellyfish blooming occurs almost every summer and has a growing trend. The blooming affects tourism, fishing, military affairs and marine sport events. Since jellyfish is not an endangered or protected species in China, so fishing for this jellyfish is permitted by the department of fisheries. Samples of the jellyfish *N*. *nomurai* were collected from the First Bathing Beach of Qingdao (36°03′25.21″ N, 120°20′11.76″ E), Shandong Province in August 2013. After capture, four entire living specimens were immediately shipped to laboratory in box. Sample of Umbrella (U), Tentacles (T) and Gonads (G) were excised manually from two living individuals and placed into sterilized 500 ml plastic zip-lock bags at 4°C for immediate use.

### Fungi Isolation

For fungi isolation, separate parts of jellyfish were rinsed in 5 successive 500 ml beakers containing 250 ml sterile natural seawater to remove contaminants from surrounding water and microorganisms attached on the jellyfish surface. Two different inoculation methods were used to cultivate jellyfish-derived fungi. For sample U, cubes of 0.5-cm^3^ were cut from the inner parts of jellyfish exumbrella and then placed gently on PDA medium (Potato Dextrose Agar medium containing 10 g sea salts, 20 g dextrose, 20 g agar powder and 1 L 20% (w/v) potato soup, natural pH). In addition, jellyfish subumbrella was sampled with sterilized forceps and cut into pieces and spread on the same medium surface after disinfecting with a cotton swab dipped in 75% ethanol. For sample T and G, about 1 g of each sample was added to 4 ml sterile natural seawater and thoroughly homogenized in a glass dounce homogenizer. A 10-fold dilution series was made and two dilutions (1:10 and 1:100) were used to inoculate the isolation media. Benzylpenicillin and streptomycin were added to each PDA medium at a final concentration of 50 μg/ml for each. All above samples were plated in triplicate. The isolation plates were incubated at 28°C for 1~3 weeks. Colonies from PDA plates were selected based on different colony morphology. Fugal isolates were obtained in pure culture after successive transfers to fresh isolation media. And the pure cultures were incubated on Wickerham’ medium (3 g yeast extract, 3 g malt extract, 5 g peptone, 10 g glucose in 1 L 75% natural seawater, 18 g agar, pH 7.2~7.4) slants at 4°C for short-term storage.

### Genomic DNA extraction, PCR amplification of rDNA-ITS fragment and 26S rDNA D1/D2 region

For DNA extraction, all fungal isolates were cultured on the Wickerham’ medium at 28°C for 7 days. The mycelia were picked and transferred to a 1.5 ml Eppendorf tube containing 50 microlitre of TaKaRa Lysis Buffer for Microorganism to Direct PCR (Code No.9164, Dalian, China). The mixture was incubated at 80°C for 15 min. then the mixture was centrifugated at 10,000 g for 5 min and the supernatant was gently transferred to a new tube as DNA templates. TaKaRa Fungi Identification PCR Kit (Code NO.RR178, Dalian, China) was used to amplify fungal ITS fragment. The reaction mixture for PCR amplification contained 1 μL of DNA templates, 25 μL PCR Premix (TaKaRa), 0.5 μL ITS Forward primer (20 pmol/μL), 0.5 μL ITS Reverse primer (20 pmol/μL) and 25 μL dH_2_O. The PCR amplifications were carried out using an initial denaturation step of 5 min at 94°C, followed by 30 cycles of 0.5 min at 94°C for denaturation, 0.5 min at 55°C for annealing, and 1 min at 72°C for extension, with a final extension cycle of 5 min at 72°C. For a fungal isolate failing to amplify ITS fragment, TaKaRa Fungi Identification PCR Kit (Code No.RR178, Dalian, China) was used to amplify the 26S rDNA D1/D2 region. PCR was performed in reaction mixtures containing1 μL of DNA templates, 25 μL PCR Premix (TaKaRa), 0.5 μL D1/D2 Forward primer (20 pmol/μL), 0.5 μL D1/D2 Reverse primer (20 pmol/μL) and 25 μL dH_2_O. PCR conditions included an initial denaturation at 94°C for 5 min followed by 32 cycles of denaturation at 94°C for 0.5 min, annealing at 55°C for 0.5 min, and elongation at 72°C for 1 min, with a final elongation at 72°C for 5 min. PCR products were purified using the Takara MiniBEST Agarose Gel DNA Extraction Kit Ver.3.0 (Code No.9762, Dalian, China) and sequenced in TaKaRa Biotechnology (Dalian) Co., Ltd.

### Nucleotide Sequences and Phylogenetic Analysis

DNA sequences were compared to the sequences within the NCBI database using the BLASTN algorithm and deposited in GenBank under the accession numbers: KP081770~KP081774. To reconstruct the phylogenetic tree, another five relative sequences from four identified jellyfish-derived fungi, *Epicoccum purpurascens* [19], *Phoma* sp. [47], *Aspergillus fumigatus* [48] and *Paecilomyces variotii* [17], were used. These five ITS sequences were FR822845, GQ352490 [18], KH00184 [18], JX022941 and JN8509969, respectively. The five sequences used for comparison were chosen based on either the literature data or the blast results of isolates from similar isolation source. Strain SmU02, identified by 26S D1/D2 region, share 99% similarity with *Purpureocillium lilacinum* strain M3748. Therefore, the ITS sequence of *Purpureocillium lilacinum* strain M3748 were used in this study. Other reference sequences were selected according to the nBLAST results. The fungus *Batrachochytrium dendrobatidis* (JQ582939) served as outgroup taxon. In conclusion, a total of 40 related sequences were used for phylogenetic analysis and their detailed information were given in S1 Table. All the 40 chosen sequences were aligned using MAFFT’s auto option [49] and further manually edited in MEGA 6.06 [50] prior to analysis. Then the data set were analyzed using maximum likelihood in MEGA 6.06 to determine the best suited nucleotide substitution model based Bayesian Information Criterion (BIC). The neighbor-joining analyses was performed in MEGA 6.06 using K2+G model of evolution as determined before, followed by bootstrap analysis with 1000 replicates.

### Small-scale fermentation and extraction

Pure strains of five jellyfish-derived fungi were grown on petri dishes containing Wickerham’ medium for seven days at 28°C. For small-scale fermentation, agar plugs (Φ = 5mm) containing fungal mycelium were punched on the seven-days-old cultures. Liquid Wickerham’s Broth (WMB: 3 g yeast extract, 3 g malt extract, 5 g peptone, 10 g glucose in 1 L 75% natural seawater, pH 7.2~7.4) and solid rice medium(RM:100 g rice, 110 ml 100% seawater containing 0.3% peptone) were used for each fungal isolate. In addition, the International Streptomyces Project Medium 2 broth (ISP2: consisting of 10 g malt extract, 4 g dextrose, and 4 g yeast extract in 1 L seawater) was also used for SmU03 and SmU05. All above media were sterilized at 121°C for 20 min. The OSMAC approach was achieved by inoculating agar plugs from each plated culture into 4 × 500 ml Erlenmeyer flasks containing 200 ml WMB or ISP2 (for liquid fermentation) or 2 × 1000 ml Erlenmeyer flasks (for solid fermentation) containing 100 g rice. For co-cultivation of the two antagonistic fungi SmU05 and SmT07 in liquid and solid medium, agar plugs of strain SmT07 were inoculated into the media and grown at 28°C. After 48h, agar plugs of strain SmU05 were inoculated into the SmT07 cultures. All the cultures were allowed to grow under static conditions at 28°C for 4~6 weeks.

At the end of the fermentation, 150 ml ~ 250ml ethyl acetate (EtOAc) were added into each culture flask and the culture flasks were left closed for another 48h. Then, the liquid cultures were separated from the mycelia by filtration and extracted with EtOAc (3 × 250 ml). The organic layers were collected and dried over anhydrous MgSO_4_, then concentrated under reduced pressure to afford the crude extracts. For solid rice media, the cultures were crushed mechanically and then extracted with EtOAc (3 × 500 ml) in an Ultraturrax (JY92-II, Scientz) at 600W for 2 × 90 cycles of running for 20 second with a 5 second internal. The homogenate was subject to filtration with medical gauze and the organic layer was collected. The combined EtOAc extract was evaporated to dryness under reduced pressure to give a crude gum. A total of 13 EtOAc extracts were obtained.

### HPLC analysis of EtOAc extracts from jellyfish-derived fungi

A milligram aliquot of the EtOAc extracts was dissolved in MeOH and then filtered through a 0.22 μm membrane filter. 20 μL of these EtOAc extracts were then subjected to HPLC analysis on an Agilent Zorbax SB-C18 column (5 μm, 150mm × 4.6mm i.d.) at a flow rate of 1.0 ml/min. The SHIMADZU Prominence HPLC system consisted of a binary LC-20AT pump, a CBM-20A communication bus module, a manual injection system, a SPD-M20A detector, a CTO-20A oven and a LC-Solution workshop. The elution gradient (Methanol-H_2_O) was set up for solvent B (methanol) as 0~5 min, 5%; 5~65 min, 5%~100%; 65~75 min, 100%B; 75~80 min, 100%~5%B.

### Antimicrobial activity

Two Gram-positive bacteria (*Staphylococcus aureus subsp*. *aureus* CGMCC 1.879 = CMCC 26001, *Bacillus subtilis subsp*. *subtilis* CGMCC 1.3382 = ATCC 11774), two Gram-negative bacteria (*Escherichia coli* CGMCC 1.872 = CMCC 44568, *Salmonella enterica subsp*. *enterica* CGMCC 1.10754 = ATCC 19585), two marine fish pathogens(*Vibrio parahaemolytic* CGMCC 1.1616 = ATCC 33847, *Listonella pelagia* CGMCC = ATCC 33783), one yeast (*Candida albicans* CGMCC 2.4091) and one fungus (*Aspergillus niger* CGMCC 3.3928) were used as test organisms in this study.

Screening for *in vitro* antimicrobial activity of crude extracts were performed according to the Antimicrobial Susceptibility Testing Standards M2-A11 outlined by the Clinical and Laboratory Standards Institute (CLSI) with some modifications [51]. Microbial test strains, except for *A*. *niger*, were recovered from 4°C and inoculate on Mueller-Hinton Agar (MHA, 6.0 g beef extract powder, 1.5 g soluble starch,17.5 g acid digest of casein, 17.0 g agar power, pH 7.3 ± 0.1) plates overnight aerobically in 37°C incubator. Single colonies were picked and adjusted to match the 0.5 Mcfarland standard with Mueller-Hinton Broth as bacterial suspension. Suspension of *A*. *niger* was prepared by pouring 5 ml 0.85% NaCl solution (contain 0.25% Tween-80) into a 7-day-old culture on Cz medium (30 g sucrose, 3 g NaNO_3_, 0.5 g MgSO_4_ • 7H_2_O, 0.5 g KCl, FeSO_4_ • 4H_2_O, 1.0 g K_2_HPO_4_, 18 g agar power in 1 L deionized water, pH 6.0–6.5). A cotton swab was used to evenly spread both bacterial and fungal suspensions on the same medium plates as above. About 100~150 mg of OSMAC extracts were resolved in 1 ml DMSO as test sample. 4 μl of sample solution were pipetted onto sterile paper disks (5 mm diameter). Then the paper disks were placed on the surface of indicator medium plates in triplicate. After inoculation at 37°C for 18h for test bacteria and yeasts and 28°C for 72h for *A*. *niger*, inhibitory activity was determined by measuring the diameter of inhibitory zone (mm). DMSO was used as negative control. Ciprofloxacin (Sigma) and amphotericin B (Sigma) were chosen respectively as positive control for bacteria and fungi. The final results were recorded as Mean ± SD of three repeats. Data were submitted to variance analysis (ANOVA) using the SPSS software (version 18.0 for Windows, SPSS Inc., Chicago, IL, USA) and means were compared by Duncan’s multiple comparison test. Statistical differences were considered to be significant at p < 0.05.

### Antifungal activity

Four plant pathogenic fungi, *Rhizoctonia solani* CGMCC 3.28, *Verticillium dahlia* CGMCC 3.3758, *Gibberella zeae* CGMCC 3.4288 and *Botrytis cinerea* CGMCC 3.4582 were used to test the antifungal activity in this study.

Antifungal bioactivities against mycelial growth of phytopathogenic fungi were performed by the agar medium assay as described in literature [52] with minor modifications. 0.5 ml tested sample solutions (Y11-1, Y11-2 and CO-1, CO-2; Table 2) were diluted in 2% Tween-80 aqueous solution [composed of 1% DMSO] to make the final volume of 50 ml. These sample solutions were added into liquid potato dextrose agar at a final concentrations of 50, 100 and 200 μg/ml. And the agar plugs containing the fungal mycelia were inoculated onto the center of PDA plates, then the plates were placed in Mould Incubator (BMJ-400, BoXun, Shanghai, China) at 28°C. The aqueous solution [composed of 1% DMSO and 2% Tween-80] was used as a negative control and amphotericin B (final concentration, 4.5 μg/ml) as positive control. When the fungal mycelium reached the edges of the control dishes, the antifungal activities were calculated. The formula for counting the percentage of growth inhibition was shown as follows:

Inhibition Activity (%) = (1-Da/Db) × 100.

*Da* was the diameter of the growth zone in the experimental dish (mm) and *Db* was the diameter of the growth zone in the control dish (mm). The experiments were carried out in quadruplicates. Results were presented as Means ± SD of four independent measurements. Data were submitted to variance analysis (ANOVA) using the SPSS software (version 18.0 for Windows, SPSS Inc., Chicago, IL, USA) and means were compared by Duncan’s multiple comparison test. Statistical differences were considered to be significant at p < 0.05.

## Conclusions

A total of 7 fungal isolates have been isolated from the inner tissues of jellyfish *N*. *nomurai* collected from the First Bathing Beach of Qingdao, China. The inner tissue of sub-umbrella was found to be rich in fungi, which yielded 5 fungal isolates. However, inner tissues of Tentacle and Gonad, which parts were thought to host diverse endobacteria yielded no fungal strains in this study. By comparing the rDNA–ITS sequences with the closest relatives, we sorted the seven phylotypes into four different genera of two phyla. To maximize the opportunities of discovering new secondary metabolites, the OSMAC approach by variation of culture media and co-culture strategy were employed. An intriguing fungal-fungal interaction comprised of *A*. *versicolor* and *Tilletiopsis* sp. was observed to induce various metabolites, which were not detected in pure cultures. In addition, we also screened the 13 extracts from OSMAC and co-cultures for the antibacterial and antifungal activity. All of the 13 EtOAc crudes exhibited different antibacterial spectra against six bacterial pathogens, three of which showed strong to significant antibacterial activities to *S*. *aureus* and *S*. *enterica*. Extracts of pure culture of *A*. *versicolor* and co-cultures of *A*. *versicolor* and *Tilletiopsis* sp. in seawater-based rice medium were diverse in metabolites and showed good antifungal activities against two phytopathogenic fungi, *R*. *solani* and *B*. *cinerea*, representing a valuable source for tapping new antifungal compounds.

## Supporting Information

S1 Fig UV absorbance chromatogram of similar peaks in HPLC-PDA profiles. (DOCX)

S2 Fig UV absorbance patterns of peaks of Y11-1, Y11-2, CO-1, CO-2 at 55 min. (DOCX)

S3 Fig Representative UV chromatograms of new peaks induced by co-culture strain *A*. *versicolor* and *T*. *albescens* in rice medium. (DOCX)

S4 Fig Representative UV chromatograms of new peaks induced by co-culture strain *A*. *versicolor* and *T*. *albescens* in liquid medium. (DOCX)

S5 Fig Representative UV chromatograms of new peaks produced by *A*. *versicolor* in rice medium. (DOCX)

S6 Fig Representative UV chromatograms of new peaks produced by *A*. *versicolor* in liquid medium. (DOCX)

S1 Table GenBank accession numbers of rDNA-ITS sequences used in phylogenetic analyses. (XLSX)
