# Supplementary material for: Exploring the Antibacterial and Antifungal Potential of Jellyfish-Associated Marine Fungi by Cultivation-Dependent Approaches
Source: PLoS One. 2015 Dec 4;10(12):e0144394. doi: 10.1371/journal.pone.0144394 (PMC4670088; doi:10.1371/journal.pone.0144394)
Supplement: S1 Fig — (DOCX) [file pone.0144394.s001.docx]

**Suporting Information:**

Y11-2：t_R_ = 61.99min

Co-2：t_R_ = 61.92min

Y11-2：t_R_ = 70.65min

Co-2：t_R_ = 71.42min

Y11-2：t_R_ = 49.70min

Y11-1：t_R_ = 50.01min

**S1 Fig. UV absorbance chromatogram of similar peaks in HPLC-PDA profiles**
