# Supplementary material for: Exploring the Antibacterial and Antifungal Potential of Jellyfish-Associated Marine Fungi by Cultivation-Dependent Approaches
Source: PLoS One. 2015 Dec 4;10(12):e0144394. doi: 10.1371/journal.pone.0144394 (PMC4670088; doi:10.1371/journal.pone.0144394)
Supplement: S2 Fig — (DOCX) [file pone.0144394.s002.docx]

**Suporting Information:**

Y11-2：t_R_ = 55.47min

Co-2：t_R_ = 55.25min

Co-1：t_R_ = 56.27min

Y11-1：t_R_ = 55.16min

**S2 Fig. UV absorbance patterns of peaks of Y11-1, Y11-2, CO-1, CO-2 at 55 min**
