# Supplementary material for: Exploring the Antibacterial and Antifungal Potential of Jellyfish-Associated Marine Fungi by Cultivation-Dependent Approaches
Source: PLoS One. 2015 Dec 4;10(12):e0144394. doi: 10.1371/journal.pone.0144394 (PMC4670088; doi:10.1371/journal.pone.0144394)
Supplement: S4 Fig — (DOCX) [file pone.0144394.s004.docx]

**Supporting information:**

Peak 8

Peak 9

Peak 10

**S4 Fig. Representative UV chromatograms of new peaks induced by co-culture strain *A. versicolor* and** [***T. albescens***](http://blast.ncbi.nlm.nih.gov/Blast.cgi#alnHdr_361051980) **in liquid medium.**
