# Supplementary material for: Exploring the Antibacterial and Antifungal Potential of Jellyfish-Associated Marine Fungi by Cultivation-Dependent Approaches
Source: PLoS One. 2015 Dec 4;10(12):e0144394. doi: 10.1371/journal.pone.0144394 (PMC4670088; doi:10.1371/journal.pone.0144394)
Supplement: S5 Fig — (DOCX) [file pone.0144394.s005.docx]

**Supporting information:**

Peak 11

Peak 12

Peak 13

**S5 Fig. Representative UV chromatograms of new peaks produced by *A. versicolor* in rice medium.**
